# Buoyancy-Activated Cell Sorting Using Targeted Biotinylated Albumin Microbubbles

**DOI:** 10.1371/journal.pone.0125036

**Published:** 2015-05-20

**Authors:** Yu-Ren Liou, Yu-Hsin Wang, Chia-Ying Lee, Pai-Chi Li

**Affiliations:** 1 Graduate Institute of Biomedical Electronics and Bioinformatics, National Taiwan University, Taipei, Taiwan; 2 Department of Electrical Engineering, National Taiwan University, Taipei, Taiwan; The Ohio State University, UNITED STATES

## Abstract

Cell analysis often requires the isolation of certain cell types. Various isolation methods have been applied to cell sorting, including florescence-activated cell sorting and magnetic-activated cell sorting. However, these conventional approaches involve exerting mechanical forces on the cells, thus risking cell damage. In this study we applied a novel isolation method called buoyancy-activated cell sorting, which involves using biotinylated albumin microbubbles (biotin-MBs) conjugated with antibodies (i.e., targeted biotin-MBs). Albumin MBs are widely used as contrast agents in ultrasound imaging due to their good biocompatibility and stability. For conjugating antibodies, biotin is conjugated onto the albumin MB shell via covalent bonds and the biotinylated antibodies are conjugated using an avidin-biotin system. The albumin microbubbles had a mean diameter of 2μm with a polydispersity index of 0.16. For cell separation, the MDA-MB-231 cells are incubated with the targeted biotin-MBs conjugated with anti-CD44 for 10 min, centrifuged at 10g for 1 min, and then allowed 1 hour at 4°C for separation. The results indicate that targeted biotin-MBs conjugated with anti-CD44 antibodies can be used to separate MDA-MB-231 breast cancer cells; more than 90% of the cells were collected in the MB layer when the ratio of the MBs to cells was higher than 70:1. Furthermore, we found that the separating efficiency was higher for targeted biotin-MBs than for targeted avidin-incorporated albumin MBs (avidin-MBs), which is the most common way to make targeted albumin MBs. We also demonstrated that the recovery rate of targeted biotin-MBs was up to 88% and the sorting purity was higher than 84% for a a heterogenous cell population containing MDA-MB-231 cells (CD44^+^) and MDA-MB-453 cells (CD44^–^), which are classified as basal-like breast cancer cells and luminal breast cancer cells, respectively. Knowing that the CD44^+^ is a commonly used cancer-stem-cell biomarker, our targeted biotin-MBs could be a potent tool to sort cancer stem cells from dissected tumor tissue for use in preclinical experiments and clinical trials.

## Introduction

Isolating a specific cell type from a mixture of cells is typically the first step in cell analysis and examination, such as isolating circulating tumor cells from blood cells and cancer stem cells (CSCs) from primary tumor cells [1]. The use of cell isolation tools is fundamental to understanding biological mechanisms and constructing reliable models of biological systems. The various cell isolation methods that are available are mostly based on density gradient, particle size, adherence, absorbance, dielectric properties, chemoresistance, and antibody binding…etc [2–4]. Above all, the antibody-binding methodology relies on the antigen-antibody recognition system of cell-surface biomarkers, and therefore provides precise sorting, such as in fluorescence-activated cell sorting (FACS) and magnetic-activated cell sorting (MACS) [5–7]. Although FACS and MACS are two major tools currently used for cell sorting, they have inherent disadvantages. FACS requires an expensive and large instrument for use in laboratory work, and is slow and also not ready for clinical cell-sorting applications. While MACS is simpler, faster, and more inexpensive than FACS, exerting a magnetic force may damage some types of cell [8]. Some other methods have been developed to speed up the sorting process and to make the instrument more compact. For example, microfluidic devices are a booming field for cell sorting on a micro scale [9–11]. However, microfluidic approaches exert substantial shear stresses on the cells, thus risking cell damage [12, 13]. A novel isolation method based on the buoyancy of the microbubbles (MBs), known as buoyancy-activated cell sorting (BACS), is reported to be a simple way to isolate specific cells [14]. Furthermore, the shear stress from a rising bubble and the tension from the buoyancy force are both far below the threshold for cell damage [15, 16].

There are some reports on the use of glass MBs or lipid MBs for BACS [14, 16, 17]. The hypothesis tested in the present study is that biotinylated albumin MBs (biotin-MBs) conjugated with the avidin linkers and biotinylated antibodies (i.e., targeted biotin-MBs) can be used for BACS. Gas-filled MBs have been used clinically as ultrasound contrast agents and for other applications, such as delivering drugs or genes into cells or for breaching the blood–brain barrier [18, 19]. Albumin MBs have inherent advantages, such as stability, simplicity of formulation, and biocompatibility [19]. Labeling the MBs with antibodies to specific molecular biomarkers—to produce so-called targeted biotin-MBs—makes either ultrasound imaging or drug delivery more efficient [20, 21]. The most common way to make targeted albumin MBs is to incorporate the avidin into the albumin MB shell, which serves as the anchor for the conjugation of biotinylated antibodies. However, the avidin and the albumin MB shell are connected by noncovalent bonds, which are much weaker than covalent bonds [22–25]. Therefore, we propose that the incorporation of conjugated biotin onto the albumin MB shell could covalently strengthen the interaction between the albumin MB shell and the antibodies. Specifically, biotin can be first conjugated to albumin by a covalent amide bond for biotin-MBs, followed by incubation with avidin and biotinylated antibodies to produce the targeted biotin-MBs.

Since intratumor heterogeneity is a major clinical problem of cancer therapies, the current study focused on BACS based on targeted albumin MBs to isolate different tumor cell subtypes. For example, MDA-MB-453 tumors (which are luminal breast cancer tumors) and MDA-MB-231 tumors (which are basal-like breast cancer tumors) express different biomarkers and multiply via different signaling pathways, and therefore should be treated with different therapeutic methods. Basal-like breast cancer tumors are known to be abundant in CD44^+^/CD24^–^ cells and exhibit stem-cell-like characteristics. In contrast, luminal breast cancer tumors mostly consist of CD44^–^/CD24^+^ cells and are thought be more differentiated [26]. Therefore, a CD44 ligand on the cell surface was chosen in this study as the molecular probe. We demonstrate that MDA-MB-231 breast cancer cells can be targeted and separated by anti-CD44-antibody-conjugated biotin-MBs. The separating efficiency was found to depend on the ratio of targeted biotin-MBs to cells, and was higher than that of targeted avidin-incorporated albumin MBs (targeted avidin-MBs). In contrast, nontargeted biotin-MBs failed to suspend the MDA-MB-231 cells. Finally, we mixed MDA-MB-231 and MDA-MB-453 cells to form a heterogenous cell population, and successfully sorted the MDA-MB-231 cells from the mixture.

## Materials and Methods

### A. Preparation of targeted biotin-MBs

**Fig 1:**
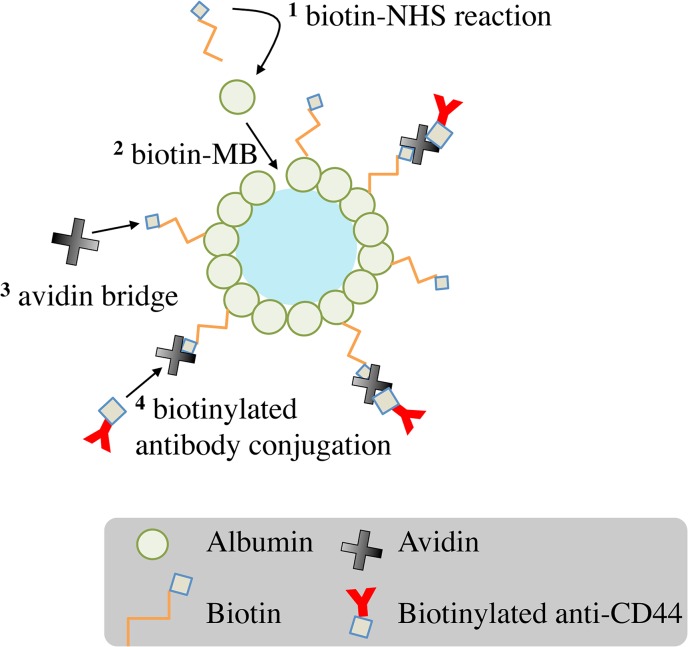
Scheme for preparing targeted biotin-MBs. The numbers represent the reaction order.

The antibodies were conjugated onto the albumin MB shell using an avidin-biotin system, as shown as Fig 1. In brief, biotin-NHS ester (Sigma-Aldrich, St. Louis, MO, USA) in dimethyl sulfoxide at 20 mg/mL was added to 10 mg/mL human serum albumin (HuSA; Octapharma, Vienna, Austria) in phosphate-buffered saline (PBS, pH = 8.0). The components were incubated on a shaker for 2 hours at room temperature and purified using a desalting column (PD-10, GE Healthcare Life Science, Buckinghamshire, UK) to prepare biotinylated HuSA. Biotin-MBs were prepared by a sonication method. A solution containing 0.33% biotinylated HuSA (w/v), 0.99% unconjugated HuSA (w/v), 5% dextrose (Sigma-Aldrich) (w/v), and perfluorocarbon (C_3_F_8_) gas to a final volume of 5 mL was sonicated by a digital sonicator (Model 450, Branson, Danbury, CT) at 200 W for 2 min [27, 28]. The freshly prepared biotin-MBs were cooled on ice for at least 2 hours. To conjugate the antibodies, 5mL biotin-MBs were incubated with 1mL avidin (2 mg/mL) overnight at 4°C and then incubated with biotinylated anti-CD44 antibodies (1:10; eBioscience, San Diego, CA) for at least 2 hours at 4°C. Control MBs were produced by sonicating the solution containing 1.32% unconjugated HuSA (w/v), 5% dextrose (w/v), and C_3_F_8_ gas in a final volume of 5 mL. Avidin-MBs were produced by adding 2 mg of avidin, 1.2% unconjugated HuSA (w/v), and 5% dextrose (w/v) to the solution prior to the sonication. The avidin-MBs were then incubated with biotinylated anti-CD44 antibodies to produce targeted avidin-MBs.

### B. Characteristics of biotin-MBs

The biotinylated HuSA was quantified using a Pierce Biotin Quantitation Kit (#28005, Thermo Fisher Scientific Inc., Rockford IL, USA). First, the concentration of reacted HuSA was measured by measurement of UV absorbance at 280 nm using a spectrometer (NanoDrop, Thermo Fisher Scientific Inc.). The biotinylated HuSA was then reacted with a 4'-hydroxyazobenzene-2-carboxylic acid (HABA dye)/avidin premix. The concentration of biotin was quantified based on the measured absorbance at 500 nm. Finally, we calculated the ratio of biotin to HuSA by dividing the concentration of biotin by that of HuSA.

MBs of selected sizes were stratified by centrifugation at an acceleration of 60*g* for 2 min three times [29]. The concentration and size distribution of the MBs were measured by a size-analysis instrument (Multisizer 3, Beckman Coulter, San Jose, CA) using a 30-μm-aperture probe. The biotinylation of MBs was tested by incubating the samples with streptavidin-FITC (eBioscience) for 2 hours, and evaluated using flow cytometry (FACSAria II, Becton Dickinson, San Jose, CA) and a phase-contrast fluorescence microscope (DMIRB, Leica, Wetzlar, Germany).

### C. Cell culture and cell-surface-biomarker expression

MDA-MB-231 human breast cancer cells were purchased from the American Type Culture Collection (Manassas, VA, USA) [30], while MDA-MB-453 human breast cancer cells were kindly provided by the laboratory of Dr. Min-Liang Kuo (National Taiwan University, Taipei, Taiwan) [31]. Both the MDA-MB-231 and MDA-MB-453 cells were cultured in Leibovitz’s L-15 medium supplemented with 10% heat-inactivated FBS and 1% penicillin/streptomycin at 37°C without CO_2_.

To check for cell-surface-biomarker expression, the MDA-MB-231 and MDA-MB-453 cells were fixed with 4% paraformaldehyde/PBS and incubated with antihuman/antimouse CD44 FITC (anti-CD44-FITC; 1:100 diluted; eBioscience) for 1 hour. The fluorescence intensity was evaluated using flow cytometry.

### D. Targeting to tumor cells

MDA-MB-231 and MDA-MB-453 cells were cultured in a 96-well plate at a concentration of 10^5^ cells per well. After being allowed to spread for 24 hours, the cells were treated with 50 μL of targeted biotin-MBs or control MBs added into each well. After 15 min of incubation, the unbound MBs were washed three times. The binding efficacy was evaluated with the aid of a bright-field microscope. Subsequently, the MBs were stained with indocyanine green (ICG; Sigma) for quantifying the binding efficacy. Two milligrams of ICG was added into the solution before sonication to label the HuSA, and the ICG-stained MBs exhibit a peak absorption at about 800 nm. The cells were treated with 50 μL of ICG-stained targeted biotin-MBs or control MBs for 15 min and then washed three times to remove the unbound MBs. The MBs binding efficacy was measured based on the absorbance at 800 nm as measured with a plate reader (EnSpire Multimode Plate Reader, Perkin Elmer, Waltham, MA, USA).

### E. Cancer-cell-separating from a single-cell-type cell suspension

For the cell-separating experiment, the MDA-MB-231 cells (or MDA-MB-453 cells) were detached with 0.25% trypsin-EDTA and resuspended in 0.18% EDTA/PBS (w/v) at a concentration of about 3×10^6^ cells/mL. The following types of MBs were included in different groups: control MBs, control MBs preincubated with avidin and biotinylated anti-CD44 antibodies, biotin-MBs alone, and targeted biotin-MBs. A mixture containing 500 μL of cell solution and MBs (at a ratio of MBs to cells of about 100:1) was well mixed for 10 min on a shaker, centrifuged at 10*g* for 1 min, and then allowed to separate for 1 hour at 4°C.

For testing the separating efficiency, 500 μL of the cell solution was mixed with different volumes of targeted biotin-MBs (0, 5, 25, 50, 100, and 200 μL), and subsequent separating steps as described above were applied. The MB layer was carefully harvested into an Eppendorf tube, and the volumes of the collected cell suspension and depleted solution were adjusted to 500 μL. The cell concentrations of the collected and depleted cells were counted with a hemocytometer after trypan blue staining.

### F. Cancer-cell-sorting from a heterogenous-cell-population mixture

For sorting the target cells from a heterogenous population mixture, the MDA-MB-231 cells were first labeled by staining the nuclei with 4’,6’-diamidino-2-phenylindole (DAPI) dye for 15 min at 37°C. DAPI can pass through the cell membrane and bind strongly to DNA in both live and fixed cells. Its blue emission is strong and can be detected effectively with the aid of a fluorescence microscope. After being carefully washed three times, the MDA-MB-231 cells were detached and mixed with MDA-MB-453 cells at a ratio of 1:4. The targeted biotin-MBs were added to the mixture with the MBs and cells at a ratio of 100:1. The subsequent sorting process was similar to that described above. The MB layer was harvested into a new Eppendorf tube and washed once. The collected cells and depleted cells were mounted onto the glass slide and observed under the fluorescence microscope (DMIRB, Leica) under the same acquisition conditions. The number of cells was counted using ImageJ software with the same color threshold.

## Results

### Characteristics of biotin-MBs

**Fig 2:**
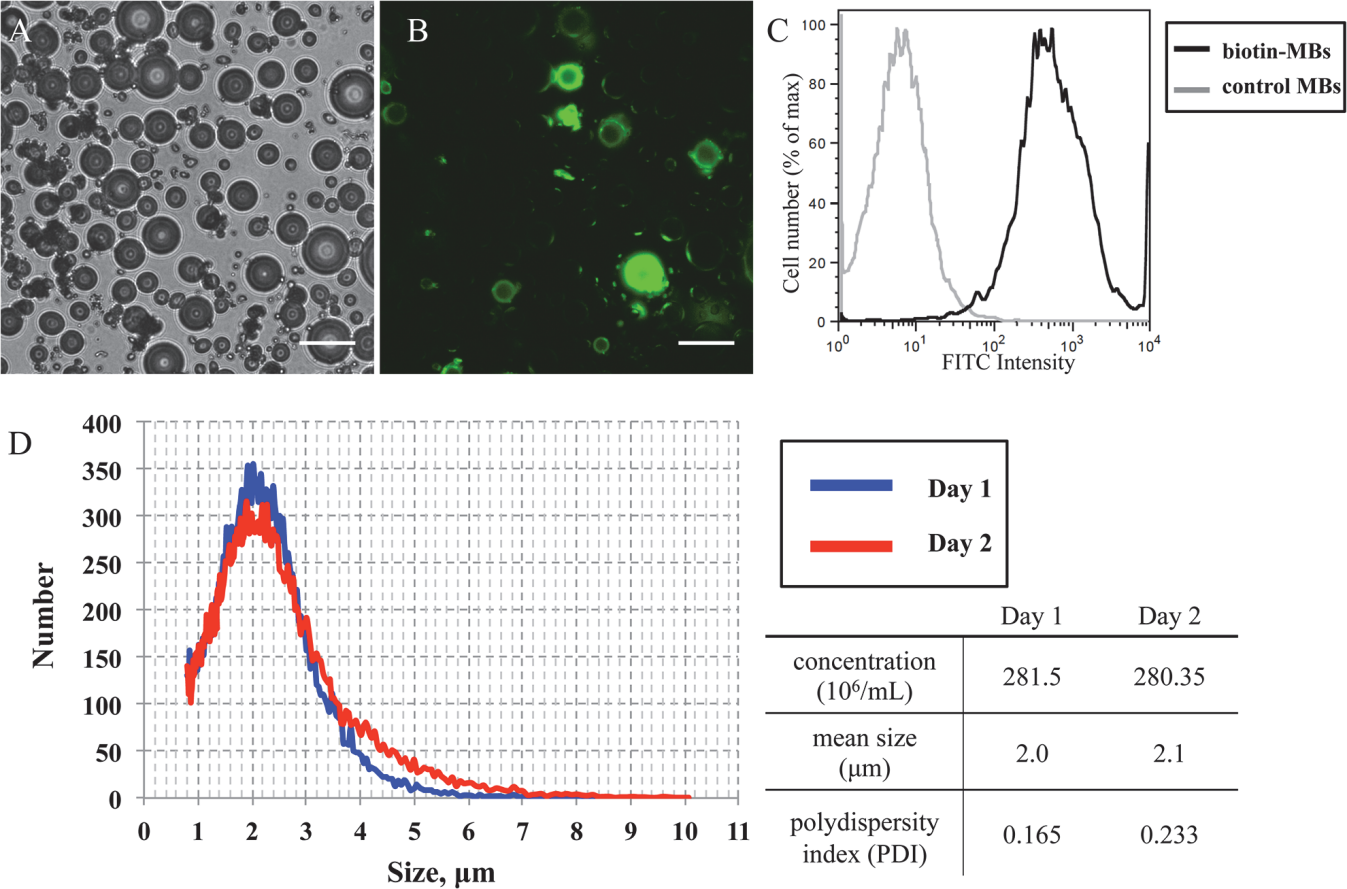
Characteristics of biotin-MBs. (A) Biotin-MBs in bright-field microscopy. (B) Biotin-MBs stained with streptavidin-FITC (green). Scale bars = 25 μm. (C) Quantification of biotinylation of the MBs (mean intensity of control MBs = 8.51; mean intensity of biotin-MBs = 1136). (D) MB size distribution over two consecutive days. The blue line represents Day 1 when MBs were fresh prepared, and the red line represents one day after MB preparation. The table shows the quantification of MB concentration, mean size, and polydispersity index (PDI).

In the first step of the method used to prepare targeted biotin-MBs, the biotin molecules were first conjugated to the HuSA with covalent bonds by a biotin-NHS ester reaction. The ratio of biotin to albumin was measured after conjugating biotin to albumin, which indicated the molar ratio of biotin molecules to HuSA molecules was 0.6~0.7:1. Biotinylated HuSA was used to make MBs via the sonication method described above. The conjugation of biotin onto the albumin MBs was tested with streptavidin-FITC by flow cytometry and fluorescence microscopy. The fluorescence image in Fig 2B shows that the streptavidin-FITC bound to the shell of the biotin-MBs. An equivalent number of unconjugated albumin MBs mixed with the same volume of streptavidin-FITC was used as the control. The results for flow cytometry also show that the fluorescence intensity was higher for biotin-MBs than for the control ones (Fig 2C). These results indicate that the biotin was successfully conjugated onto the shell of the MBs. Furthermore, the concentration, size, and the polydispersity index (PDI, defined as the square ratio of the standard deviation and mean diameter) were measured over two consecutive days. The results show that the biotin-MBs were stable over that time period, with a concentration at about 280×10^6^/mL and a mean size of 2 μm with PDI of 0.165 and 0.233. (Fig 2D).

### Targeted biotin-MBs attach to target cells

**Fig 3:**
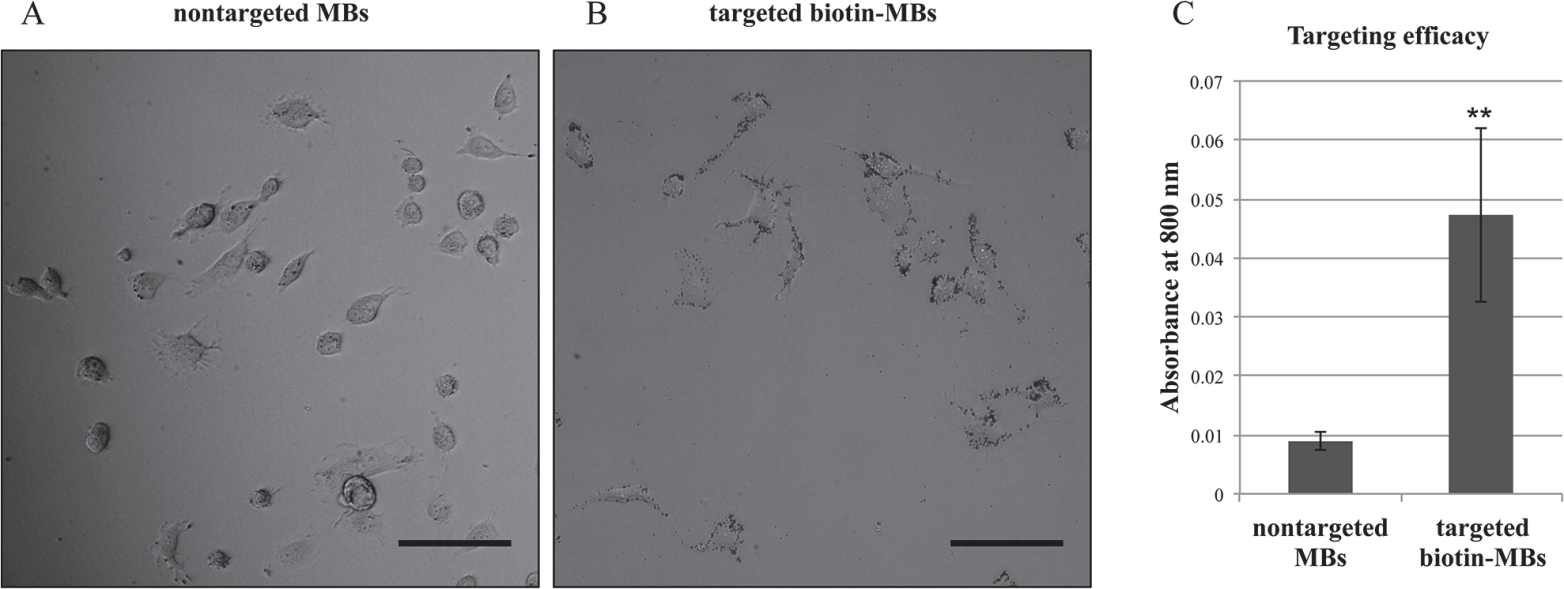
Targeted biotin-MBs conjugated with anti-CD44 antibodies can recognize and bind to MDA-MB-231 cells. (A) MDA-MB-231 cells treated with nontargeted biotin-MBs in bright-field microscopy. (B) MDA-MB-231 cells treated with targeted biotin-MBs. Scale bars = 100 μm. (C) Comparison of targeting efficacy with ICG-stained MBs. Data are mean and SD values. (* represents t value < 0.05, and ** represents t value < 0.001).

In order to bind to the target cells (MDA-MB-231 cells), the biotin-MBs were further conjugated with avidin, followed by biotinylated anti-CD44 antibodies. Fig 3A and 3B show the images of MDA-MB-231 cells after a 15-min incubation with control MBs or targeted biotin-MBs, respectively. The MDA-MB-231 cells are observed to bind to abundant targeted biotin-MBs (Fig 3B) but few control MBs (Fig 3A). Both the targeted biotin-MBs and control MBs were stained with ICG to allow a quantitative comparison of the binding efficacy. The ICG-stained MBs have a peak absorption at 800 nm, and hence the absorbance at 800 nm can be used to evaluate the binding of MBs to the cells at high specificity. Fig 3C shows that the absorbance at 800 nm of the MDA-MB-231 cells treated with targeted biotin-MBs was five to six times higher than that for the control nontargeted biotin-MBs (n = 5). This confirms that the anti-CD44-antibody-conjugated biotin-MBs can recognize and attach to the target MDA-MB-231 cells.

### Separating breast cancer cells using targeted biotin-MBs

**Fig 4:**
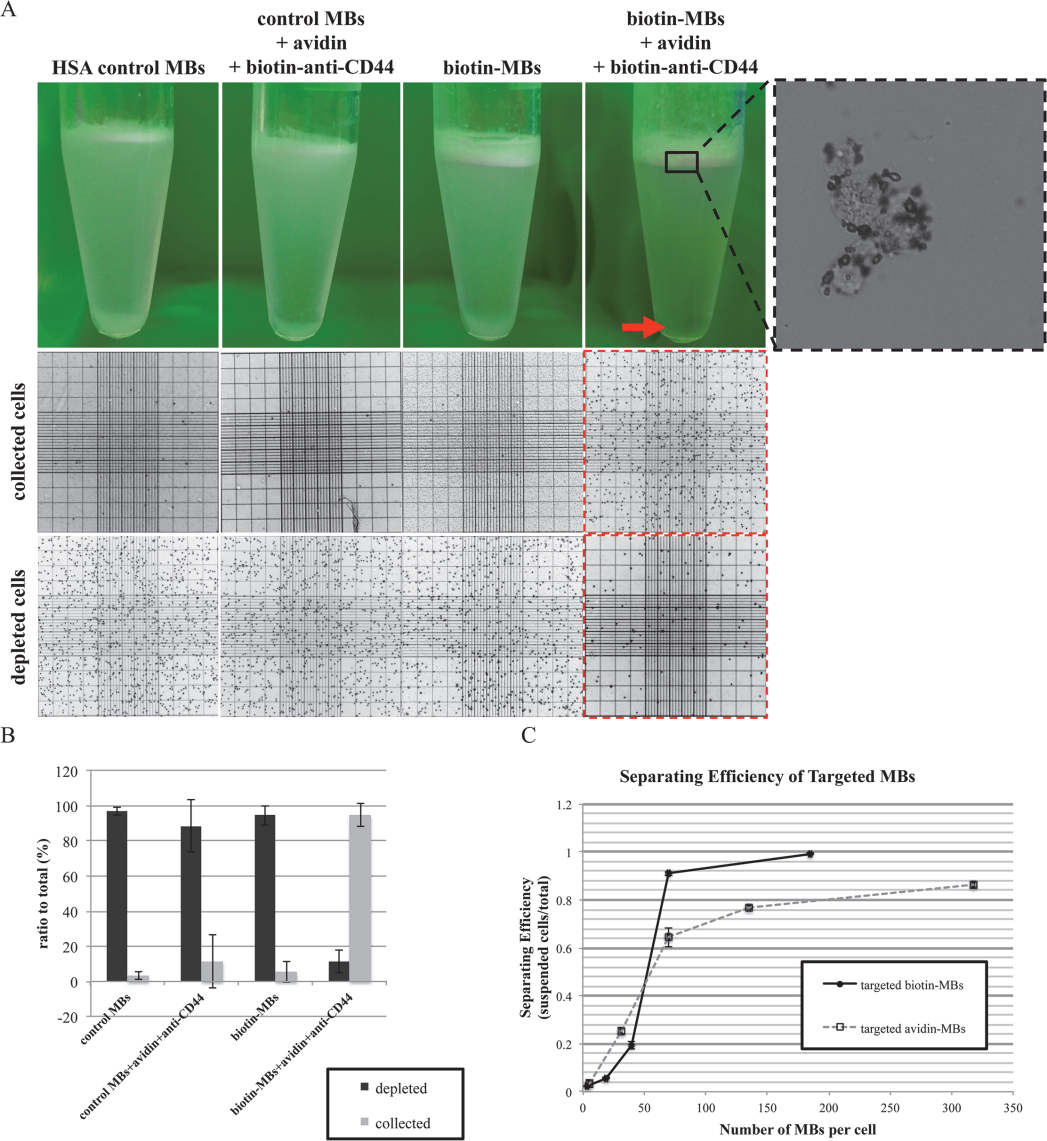
Using the targeted biotin-MBs for cell separating. (A) Comparison the separating abilities of targeted biotin-MBs, nontargeted biotin-MBs, and control MBs. Only the MDA-MB-231 cells with targeted biotin-MBs were suspended, in which most of the cells were collected in the MB layer. The cell concentrations of the collected and depleted cells were counted with a hemocytometer after trypan blue staining. Red arrow indicates the depleted layer. (B) Quantification of cells in the collected and depleted layers. (C) Relationship between separating efficiency and the ratio of targeted biotin-MBs to cells (black filled circles and black hollow squares represent the targeted biotin-MBs and targeted avidin-MBs, respectively). (D) Microscopy images of collected cells. Targeted biotin-MBs are observed binding to the MDA-MB-231 cells. Scale bar = 50 μm.

To test the use of targeted biotin-MBs in cell separating from the single-cell-type cell suspension, the target MDA-MB-231 cells were mixed with control MBs, control MBs preincubated with avidin and biotinylated anti-CD44 antibodies, biotin-MBs, or targeted biotin-MBs (the ratio of MBs to cells was about 100:1 in all cases). Fig 4A shows that only the targeted biotin-MBs could be used for separating the MDA-MB-231 cells—the cells almost disappeared from the depleted layer and 94.6% of the cells were lifted up with the MBs and collected in the MB layer (Fig 4A, red arrow for the depleted layer and red dashed squares for both layers; and Fig 4B, n = 4). In contrast, control MBs (with or without avidin and biotinylated anti-CD44 antibodies) and biotin-MBs without antibody conjugation, all failed to lift up and separate the cells. This indicates that our targeted biotin-MBs could sort the cells successfully, and also confirms minimal non-specific adhesion between the MBs and the cells.

The separating efficiency was quantified with different volumes of targeted biotin-MBs. The separating efficiency was calculated as the number of collected cells divided by the total number of cells (i.e., collected plus depleted). The black filled circles in Fig 4C show that the number of separated cells increased with the ratio of MBs to cells. More than 90% of the cells could be separated for a volume of targeted biotin-MBs of 100 μL and a corresponding ratio of MBs to cells of 70:1 (n = 2). A higher efficiency of 99.2% was achieved when the ratio of MBs to cells was increased to 185:1. We also compared the targeted biotin-MBs with the targeted avidin-MBs, with the results showing that the separating efficiency was 13% lower for the targeted avidin-MBs than for the biotin-conjugated MBs at MB to cell ratio greater than 70:1 (Fig 4C, black hollow squares). Fig 4D shows microscopy images of the collected cells, which surrounded by the targeted biotin-MBs.

### Sorting of target cells from a heterogenous-cell-population mixture

**Fig 5:**
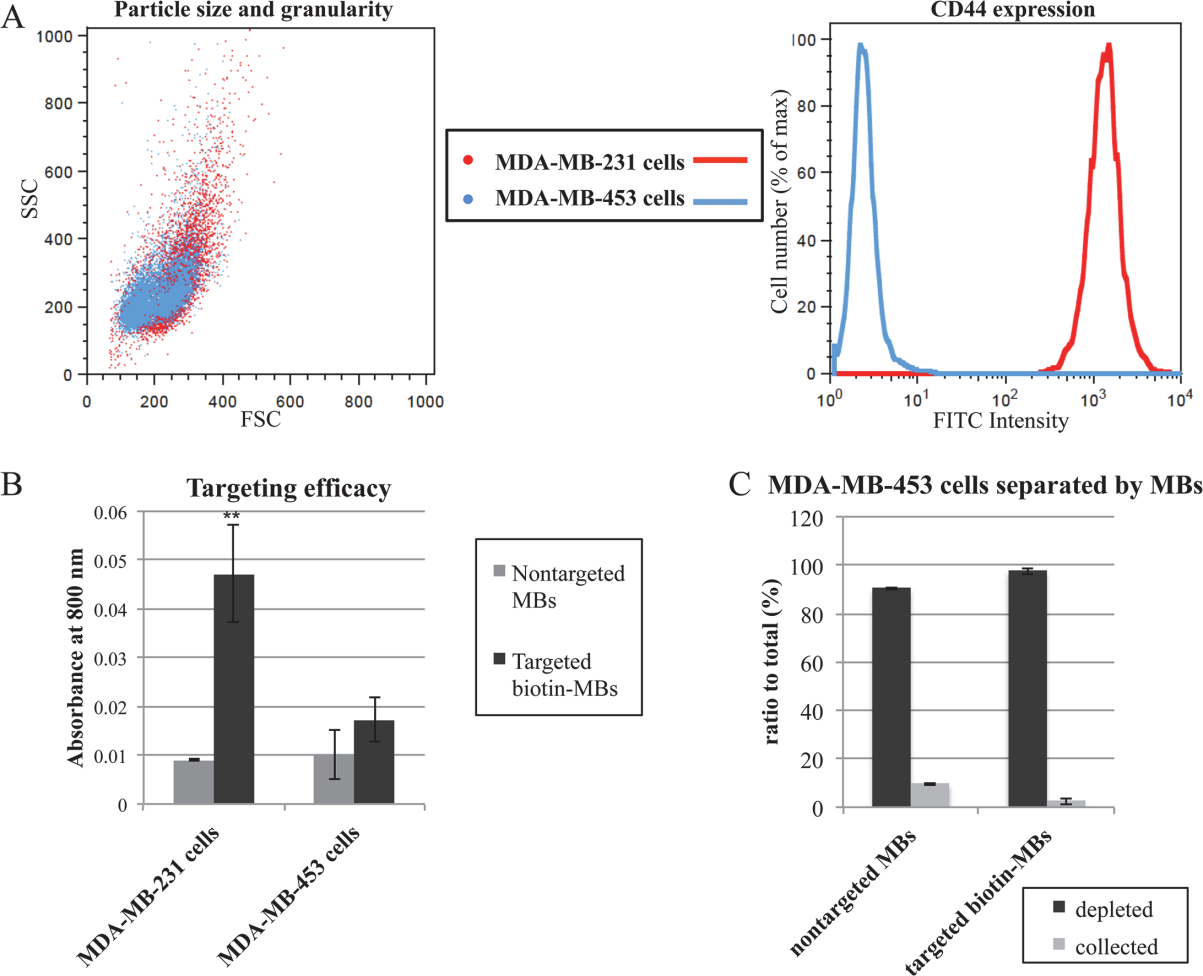
Targeted biotin-MBs cannot separate the CD44-deficient MDA-MB-453 cell population. (A) The particle size, granularity and CD44 expression of MDA-MB-231 and MDA-MB-453 cells as determined using flow cytometry. Red and blue dots/lines represent MDA-MB-231 and MDA-MB-453 cells, respectively. (B) Comparison of targeting efficacy of targeted biotin-MBs to MDA-MB-231 and MDA-MB-453 cells with ICG-stained MBs. Data are mean and SD values. (* represents t value < 0.05, and ** represents t value < 0.001). (C) Using the targeted biotin-MBs and control for separating the MDA-MB-453 cells in the cell suspension and quantifying the cell concentrations of the collected and depleted layers.

To further evaluate the performance of the targeted biotin-MBs in cell sorting, we added another cell type (MDA-MB-453 cells) to the cell suspensions. We first tested the expression of CD44 on MDA-MB-453 cells, and compared the results with those for MDA-MB-231 cells. The MDA-MB-231 and MDA-MB-453 cells were stained with anti-CD44-FITC, and then the FITC intensity was measured using flow cytometry. The results showed that while the particle sizes and granularity of the two cells were similar (where the sizes and granularity of MDA-MB-231 cells were slightly more dispersive than those of MDA-MB-453 cells), the expression level of CD44 was much higher for MDA-MB-231 cells than for MDA-MB-453 cells (Fig 5A). Next, the targeted biotin-MBs stained with ICG were used to evaluate the targeting efficacy on MDA-MB-453 cells. After 15 min of incubation the absorbance at 800 nm did not differ significantly irrespective of whether the MDA-MB-453 cells were treated with control MBs or targeted biotin-MBs (Fig 5B, n = 5). Furthermore, the control MBs and targeted biotin-MBs failed to separate the MDA-MB-453 cells (Fig 5C, n = 2). This result supports that MDA-MB-453 cells can be used in sorting experiments for distinguishing these cells from MDA-MB-231 cells when both cell types are present in a heterogenous-cell-population mixture.

**Fig 6:**
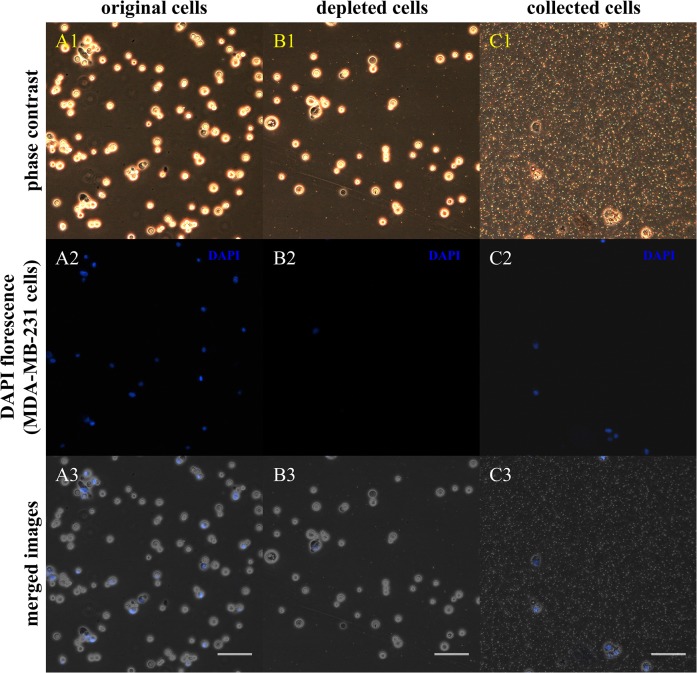
Sorting of MDA-MB-231 cells from cell mixtures. The images show the compositions of cell mixtures before sorting (A1, A2, and A3), depleted cell suspensions after cell sorting (B1, B2, and B3), and the collected MB layer (C1, C2, and C3). (A2, B2, and C2) DAPI staining (blue) representing MDA-MB-231 cells. (A3, B3, C3) Merged phase-contrast and DAPI images. Gray cells without blue overlay represent MDA-MB-453 cells (scale bar = 100 μm).

Before mixing the two cell populations, we labeled the MDA-MB-231 cells by staining their nuclei with DAPI. The DAPI-labeled MDA-MB-231 cells were mixed with MDA-MB-453 cells and treated with targeted biotin-MBs. The MB layer and the depleted solution were collected. Phase-contrast images were obtained first, followed by DAPI fluorescence images. Fig 6 shows the results, where the DAPI-labeled cells (Fig 6. A2, B2, and C2; blue staining) represent the MDA-MB-231 cells, and the other cells without DAPI staining are MDA-MB-453 cells (Fig 6. A3, B3, and C3; gray cells without blue overlay). The percentage of MDA-MB-231 cells was 20.83±4.5% in the original cell mixture and 2.33±3.5% in the depleted solution after cell sorting; that is, the sorting efficiency was 88.79±3.6% (n = 3). The suspension of collected cells comprised 84.6±5.5% MDA-MB-231 cells, which indicates the sorting purity.

## Discussion

In this study we first conjugated biotin onto the albumin MB shell with a high efficiency. The streptavidin-FITC intensity was more than one-hundred-fold higher for biotin-MBs than for control MBs. After anti-CD44 antibodies were conjugated, the targeted biotin-MBs could successfully attach to the target MDA-MB-231 breast cancer cells and be used to separate these cells from the single-cell-type suspension. Although it is easier to fabricate the commonly used avidin-MBs than the proposed biotin-MBs [20], our results show that the separating efficiency is lower for targeted avidin-MBs than for targeted biotin-MBs. One possible advantage of biotin-MBs may come from the expanded conjugation of antibodies, mitigating the steric hindrance problem when antibodies recognize the cells, thus increasing the binding efficiency [32, 33]. Another advantage may be the increased strength of the conjugation between the albumin MB shell and the antibodies due to the presence of covalent bonds between the biotin and albumin, rather than simple physical embedding of the avidin into the albumin MB shell. More measurements are required to confirm the advantage of biotin-MBs over avidin-MBs.

Different tumor cell subtypes may multiply via different signaling pathways, and therefore require different therapeutic treatments. Above all, the CSCs (or tumor-initiating cells) are thought to be related to the initialization and maintenance of the tumors, thus playing a crucial role in cancer research. Some previous studies have found that the lymphocyte-homing receptor CD44, a cell-surface biomarker, is strongly associated with tumor progression and metastasis, and is commonly used as biomarker of CSCs [34, 35]. Furthermore, CD44 expresses various isoforms in different types of tumor and exhibits different stem-cell behaviors [36, 37]. Therefore, the sorting of CD44^+^ cells may be necessary for in-depth studies in the field of CSCs. There are also some commercial products designed to sort CD44^+^ CSC cells, such as CD44 MicroBeads (Miltenyi Biotec, Bergisch Gladbach, Germany). However, there is some evidence that magnetic beads may adversely affect the characteristics of the target cells [38]. In the present study we have demonstrated the usefulness of targeted biotin-MBs in sorting the CD44^+^ MDA-MB-231 cells from the CD44^–^ MDA-MB-453 cells based on the buoyancy of MBs, which does not exert any other stimulus on the cells and is a gentle and simple way to sort target cells from a mixture. In our experiments the recovery rates reached about 88% and the purity was about 84%. Some of the nonspecific binding may have been due to the binding of albumin MBs to the DNA released from the dead cells in the sorting process or to cell–cell interactions resulting in the formation of cell clusters [2].

The results reported herein indicate that target cells can be sorted in a simple and portable way. The results reported herein indicate that target cells can be sorted in a simple and portable way. The use of glass MBs or lipid MBs for BACS have been reported to be an effective and efficient method to isolate the target cells [14, 16]. However, this is the first study in the literature using albumin MBs to separate and sort the CSCs from different tumor cell subtypes. To our knowledge, albumin MBs are shown to have inherent advantages, such as stability, simplicity of formulation, and biocompatibility, over MBs made by other materials [39]. The chemical cross-linking of albumin shell makes albumin MBs have higher rigidity and persistence during myocardial contrast echocardiography [18, 40, 41]. Therefore, albumin MBs are suitable for applying an acoustic radiation force *in vitro* or *in vivo* experiments [42–45], which can displace the contrast agents and can be used to accelerate the sorting process. Some studies have shown that the usage of cationic lipid MBs combined with the acoustic radiation force for delivery of mesenchymal stem cells to vascular endothelium [46]. Our targeting albumin MBs with improved cell targeting and MB rigidity may provide benefits for acoustic-radiation-force assisted cell-based therapy. Furthermore, we developed a simple two-step and flexible procedure to conjugate the antibodies onto the albumin MBs by the avidin-biotin system. The targeted albumin MBs can be easily modified by simply replacing the biotinylated antibodies in the last step. In addition, the MBs employed in this method can be destroyed easily by simple sonication (within 1 second) to release the sorted cells and make them available for further analysis. Based on the buoyancy of the MBs, the system may be further applied to cell suspension cultures for culturing stem cells. It can be difficult to select specific cells with multiple biomarkers simultaneously when using BACS, making this method more suitable for the bulk selection of cells than for the analysis of individual cells. However, this limitation of BACS may be at least partially overcome by employing multiple steps—because the MBs are easily removed from the cells, different cells can be released in sequential selections.

## Conclusion

The usage of targeted biotin-MBs for BACS is reported herein. The biotin is conjugated to albumin MBs with high efficiency in our fabrication process. The separating efficiency of targeted biotin-MBs to MDA-MB-231 cells is higher than 90% when the ratio of MBs to cells is 70:1. This performance is better than that of conventional targeted avidin-MBs. In sorting experiments involving cell mixtures containing MDA-MB-231 and MDA-MB-453 cells, both the recovery rate and purity of the targeted biotin-MBs were higher than 80%. These results indicate that using targeted biotin-MB for BACS may be an effective tool for cell isolation in preclinical experiments and clinical trials.
